# The contribution of imprinted genes to neurodevelopmental and neuropsychiatric disorders

**DOI:** 10.1038/s41398-022-01972-4

**Published:** 2022-05-21

**Authors:** Anthony R. Isles

**Affiliations:** MRC Centre for Neuropsychiatric Genetics and Genomics, School of Medicine, Cardiff University, Cardiff, CF24 4HQ UK

**Keywords:** Clinical genetics, Epigenetics and behaviour

## Abstract

Imprinted genes are a subset of mammalian genes that are subject to germline parent-specific epigenetic modifications leading monoallelic expression. Imprinted gene expression is particularly prevalent in the brain and it is unsurprising that mutations affecting their expression can lead to neurodevelopmental and/or neuropsychiatric disorders in humans. Here I review the evidence for this, detailing key neurodevelopmental disorders linked to imprinted gene clusters on human chromosomes 15q11-q13 and 14q32, highlighting genes and possible regulatory links between these different syndromes. Similarly, rare copy number variant mutations at imprinted clusters also provide strong links between abnormal imprinted gene expression and the predisposition to severe psychiatric illness. In addition to direct links between brain-expressed imprinted genes and neurodevelopmental and/or neuropsychiatric disorders, I outline how imprinted genes that are expressed in another tissue hotspot, the placenta, contribute indirectly to abnormal brain and behaviour. Specifically, altered nutrient provisioning or endocrine signalling by the placenta caused by abnormal expression of imprinted genes may lead to increased prevalence of neurodevelopmental and/or neuropsychiatric problems in both the offspring and the mother.

## Introduction

Epigenetics, broadly defined as the molecular mechanisms that lead to long lasting regulation of gene expression thus linking gene with phenotype, is now a major area of focus for neuroscience research, primarily thanks to technological developments over the past few decades [1]. Much of our understanding of the basic epigenetic mechanisms such as DNA methylation (DNA-me), chromatin modification and non-coding RNA, originally came from the study of a small subset of mammalian genes that are subject genomic imprinting [2]. The existence of genomic imprinting was initially recognised in the early 1980’s [3, 4], and the first so-called imprinted genes identified a few years later [5]. We now acknowledge the existence of approximately 250 canonical imprinted genes in the mouse, and 200 in humans [6].

**Fig. 1:**
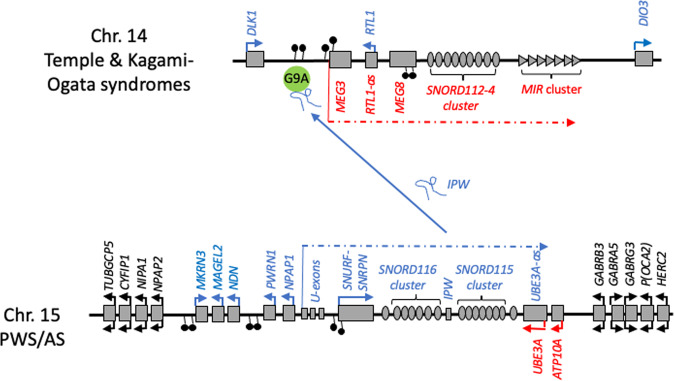
Schematic showing the imprinted gene clusters on human chromosomes 14q32 and 15q11-q13. Paternally expressed imprinted genes are represented in blue, maternally imprinted genes in red, biallelic genes in black; DNA-me is represented by “lollipops” and these define the associated DMRs (direction indicates whether methylated on paternal or maternal chromosome). Both imprinted clusters contain maternally and paternally expressed protein coding genes, snoRNA species and non-coding and/or micro-RNA molecules. The schematic also shows how the non-coding RNA *IPW* transcribed from the paternal chromosome 15 is linked to the imprinting on chromosome 14. Specifically, *IPW* is thought to link with the histone methyltransferase G9A, bind to the DMR and suppress gene expression on the paternal chromosome 14, thus leading to maternal only expression of *MEG3, MEG8*, the *SNORD112-4* and *MIR* clusters.

Canonical imprinted genes are defined by the presence of a parent-of-origin specific epigenetic mark, inherited via the germline, which ultimately leads to monoallelic expression of associated imprinted genes from one parental allele only. The initial epigenetic mark is DNA-me, and occurs at an ‘imprinting control region’ (ICR), a region of regulatory DNA that is often completely methylated on one parental copy, but un-methylated on the other (also known as ‘differentially methylated regions’, or DMRs) (Fig. 1) [7]. However, for a small number of imprinted genes, the key parent-of-origin-specific epigenetic mark has recently been shown to be histone modification, and not DNA-me [8]. Following fertilisation these parent-of-origin specific epigenetic marks are protected from genome-wide changes in DNA-me in the early embryo and are then inherited into the developing somatic cell lineages where they are maintained and added to with further DNA-me imprints [9] and changes to histones and chromatin [2]. However, in the developing germline cells, the inherited germline epigenetic imprints are erased and reset according to the sex of the embryo, and therefore whether they will pass onto to the next generation via the mother, or the father.

**Fig. 2:**
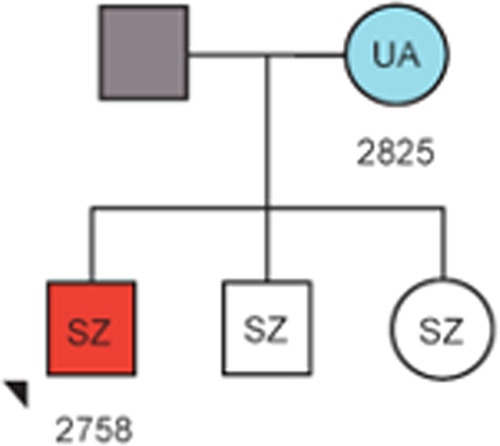
Family tree depicting transmission of 15q11-q13 duplications and neuropsychiatric phenotypes. Red fill indicates maternal duplications, blue indicates paternal duplications, and grey indicates no duplications. Samples where no DNA was available have no fill. Neuropsychiatric phenotype is indicated as follows: SZ schizophrenia, UA unaffected. In this particular pedigree the mother has inherited the duplication paternally and is (in this case) unaffected. However, when the duplication is passed through her germline the imprint is then reset and the CNV is inherited maternally in her offspring, leading to schizophrenia. Figure adapted from Isles et al. [61].

Although providing a fascinating template for understanding epigenetics mechanisms, it is the parental allele-specific monoallelic expression of imprinted genes that is key for their functional role in health and disease. Although for some imprinted genes their monoallelic status is tissue-specific, parental expression is fixed early in development, and means that some genes are only ever expressed from the paternally inherited allele, whilst others are only ever expressed from the maternal allele. As a consequence of how we think genomic imprinting evolved [10], the expression level of imprinted genes is critical, and mutations leading to deviations in expression of the active parental allele, both up and down, can have phenotypic effects. Conversely, mutations that affect the normally silenced parental allele often have no, or neutral consequences. This may lead to phenotypes skipping generations until the mutation is passed through the appropriate parental germline and is present as the active parental allele [11] (Fig. 2).

As we understand more about the functional role of different imprinted genes it is clear there are patterns to the physiologies upon which they impact [6]. Broadly, these are placental development and function; metabolism and thermoregulation; and brain and behaviour. Given their prominent role in brain and behaviour [12], it is also unsurprising that when expression of imprinted genes is perturbed in humans this can lead to neurodevelopmental and/or neuropsychiatric disorders. Here, I provide an overview of the evidence for this, but also expand the review to describe how the ‘indirect’ effect of non-CNS expressed imprinted genes may also lead to abnormal brain and behaviour.

## Imprinted neurodevelopmental disorders

A number of disorders have been explicitly linked to mutations affecting imprinted genes [13]. Most of these a caused by genetic mutations, and often by deletion copy number variants (CNVs) that span imprinted loci; deletions affecting the active parental allele effectively lead to complete loss of expression. Additionally, uniparental disomy, where both copies of a chromosome containing imprinted loci are derived from one parent, will lead to an imbalance in expression. For instance, maternal uniparental disomy (mUPD) would lead to ~2-fold increase in expression of maternally expressed imprinted genes, and a total loss of expression of paternal expressed imprinted genes. More rarely, some neurodevelopmental disorders also occur as a consequence of deletion mutations spanning the associated ICR leading to dysregulation of the imprinting at the locus. Finally there are epimutations, in which the genetic material remains intact, but the DNA-me at the ICR is lost leading to loss of imprinting. However, these epimutations are very rare and possibly most often arise as a consequences of in vitro culture, such as occurs in some instances of Assisted Reproductive Technologies [14].

Although nearly all imprinted disorders have some brain-related phenotypes [13], below I highlight some disorders with explicit neurodevelopmental and/or neurological deficits that are central features of their aetiology.

### Disorders associated with 15q11-q13

The first disorders of any kind to be associated with genomic imprinting were the neurodevelopmental disorders Angelman and Prader-Willi syndromes [15, 16]. Both are due to loss of gene expression in the chromosome 15q11-q13 interval, primarily caused by a deletion spanning the imprinted cluster (Fig. 1). However, due to the nature of genomic imprinting, the presence of this deletion can have very different phenotypic outcomes depending on whether it is present on the maternal or paternal copy of chromosome 15.

Angelman syndrome (AS) is a severe neurodevelopmental disorder, where individuals have learning difficulties with little or no speech, ataxia, seizures and EEG abnormalities. AS is usually caused by a deletion on maternal 15q11-q13, that means the expression of the two normally paternally silenced/maternally expressed imprinted genes within the cluster, *UBE3A* and *ATP10C*, is lost. However, analysis of rarer mutations indicated that the key causal imprinted gene for Angelman syndrome is in fact *UBE3A* [17, 18], which encodes the E6-AP ubiquitin ligase. Studies of this gene function in model systems has demonstrated its critical importance for neuronal development [19], experience dependent maturation, and many aspects of brain function [20–22]. Consequently, efforts to develop potential treatments for AS has focused on mechanisms for reactivating the normally silenced paternal copy of *UBE3A* in order to compensate for the loss of the deleted and/or non-functioning maternal copy [23, 24].

In contrast to AS, Prader-Willi syndrome (PWS) is primarily caused by deletion mutations affecting the paternal 15q11-q13 imprinted interval, although a number of other mutation genotypes are recognised (more later). Nevertheless, the key shared consequence of these mutations is the loss of expression of maternally silenced/paternally expressed imprinted genes, of which there are many coding and non-coding genes (see Fig. 1). Individuals with PWS are characterised by a heterogenous set physiological phenotypes, ranging from hypotonia and a poor ability to suckle at birth, growth retardation, metabolic and endocrine problems (reproductive- and feeding-related), and hyperphagia [25]. Neurodevelopmental phenotypes include mild learning disabilities, behavioural problems (tantrums, obsessive compulsive disorders including skin picking), disturbance of circadian rhythms and sleep problems, and an increase prevalence of psychotic illness [25, 26].

Comparable to the situation in AS, a number of very rare mutations have allowed some determination of key causal genes in PWS. For instance, mutations that affect the paternal copy of *MAGEL2* alone lead to Schaaf-Young syndrome (SYS) [27]. Although SYS has a great deal of overlap with PWS, pointing to the possible physiological contribution of *MAGEL2*, as more individuals have been identified it has been recognised as a separate disorder [28]. Additionally, microdeletion mutations have defined a PWS ‘critical interval’, which spans the *SNORD116* cluster and *IPW* (Fig. 1) [29, 30]. Individuals with this deletion, and indeed PWS-critical interval mouse models [31–33], show many of the phenotypes regarded as core to PWS; namely growth retardation, hyperghrelinaemia and hyperphagia. However, in contrast to the clear link between *UBE3A* and AS, whether loss of *SNORD116* and *IPW* alone lead to the full range of PWS phenotypes is less certain [34]. Animal studies of individual gene knockouts suggest that many the paternally expressed genes from the homologous 15q11-q13 imprinted gene interval contribute to the overall PWS phenotype [35]. As there is unlikely to be one “PWS-gene”, development of gene-centred therapies for PWS has been limited. Instead effort has been made to more broadly modify the epigenetic regulation of the locus as a whole [36], with some success coming from inhibiting the histone methylatransferase, G9a [37].

### Disorders associated with 14q32

Temple syndrome (TS) [38, 39] and Kagami-Ogata syndrome (KOS) [40] are caused by mutations arising with the imprinted cluster on human chromosome 14q32 (Fig. 1). Both TS and KOS are multisystem disorders, with developmental, morphological, endocrine and neurological features. Unlike AS and PWS, that are quite distinct clinically, interestingly TS and KOS have a number of overlapping clinical phenotypes despite being recognised as different disorders [41, 42].

Although both TS and KOS can be caused by a variety of deletion mutations and epimutations, the majority (>55%) are caused by chromosome 14 UPD [42]. Specifically, TS results as a consequence of mUPD, which leads to loss of maternally silence/paternally expressed genes and overexpression of paternally silenced/maternally expressed genes from the locus; KOS results as a consequence of paternal UPD (pUPD), which leads to loss of paternally silence/maternally expressed genes and overexpression of maternally silenced/paternally expressed genes from the locus.

A number of the imprinted genes within the 14q32 cluster are strongly expressed in the brain [43–45], and studies of targeted deletions of genes within homologous mouse imprinted cluster on distal chromosome 12 distal have highlighted their functional importance. Of particular prominence is maternally silenced/paternally expressed *Dlk1*, encoding the Delta-like 1 homologue, an atypical NOTCH ligand. It has been known for some time that *Dlk1* is associated with dopamine neuron differentiation [46], but more recent evidence has directly linked the imprinting status of *Dlk1* in neural stem cells (NSCs) as being a key regulator of neurogenesis [47]. Specifically, although *Dlk1* is generally expressed from the paternal allele only, biallelic expression is required in NSCs and loss of either paternal or maternal (or both) expression leads to deficits in neurogenesis and neurogenesis-dependent behaviour [48]. In addition to *Dlk1*, disruption to the paternally silenced/maternally expressed long non-coding RNA transcript, specifically deletion of the maternal copy of microRNAs miR-379/miR-410, produces animals with a specific enhancement of anxiety behaviour [49].

In light of the varied and important roles of these imprinted genes in brain and behaviour it is unsurprising that individuals with TS and KOS present with a number of neurodevelopmental/neurological phenotypes [42, 50]. Included here are neonatal hypotonia and an associated poor feeding or limited such reflex in neonates; speech and/or motor delay and learning disability; and compulsive feeding. These neurodevelopmental/neurological phenotypes are strongly reminiscent of those seen in PWS, as are many of the wider phenotypes seen in KOS and, especially, TS (Table 1) [41, 42]. This may be more than mere coincidence, as there is a regulatory link between the PWS non-coding RNA *IPW* and maternal gene expression in the imprinted cluster on 14q32 [51]. Although the mechanism has thus far only been demonstrated in human induced pluripotent stem cells (iPSCs), *IPW* forms a complex with the histone methyltransferase G9a and binds a DMR on 14q32 (Fig. 1) leading to repressive histone marks and reducing expression of maternally expressed genes (MEGs) in the interval. Loss of *IPW*, as seen in iPSCs and brain samples derived from PWS patients, leads to increased expression of MEGs on 14q32 [51]. This cross-talk between imprinted loci is reminiscent of other ‘imprinted gene networks’ [52], and suggests that part of the PWS phenotype may result as a consequence of abnormal expression from 14q32, possibly explaining the high levels of overlap with clinical feature seen in TS and KOS.

**Table 1:** Overlapping clinical features between TS, KOS and PWS.

Temple syndrome	Kagami-Ogata syndrome	Prader-Willi syndrome
IUGR/PNGR	IUGR/PNGR	IUGR/PNGR
Body asymmetry	–	Facial asymmetry in neonates
Small hands and feet	–	Small hands and feet
Hypotonia	Hypotonia	Hypotonia
Feeding problems in infancy	Feeding problems in infancy	Feeding problems in infancy
Learning disabilities	Mild learning disabilities	Learning disabilities
–	Respiratory distress	Breathing problems
Insulin resistance	–	Hypoinsulimenia and high insulin sensitivity
Early onset puberty	–	Early onset puberty
Compulsive eating habits^a^	–	Hyperphagia
Obesity	–	Obesity

A number of clinical features and/or abnormal physiologies are shared between TS/KOS and PWS (this is not a complete list of clinical features). These correlative features may be indicative of the molecular regulatory relationship between the imprinted gene clusters on chromosomes 14 and 15, specifically that *IPW* links with the histone methyltransferase G9A, binds to the DMR, and suppress gene expression on the paternal chromosome 14.

*IUGR* intrauterine growth restriction, *PNGR* post-natal growth restriction.

^a^Not consistently present in TS [50].

## Imprinted genes linked to neuropsychiatric disorders

Through the efforts of the psychiatric genetics consortium, large studies have been conducted in order to detect common genetic variation linked with neuropsychiatric disorders such as schizophrenia and bipolar disorder. These genome-wide association studies (GWAS) have been hugely successful and have identified common variation associated with hundreds of genes [53, 54]. Nevertheless, apart from a number of candidate gene-led studies [55–57], there have been limited numbers of common variants linked to imprinted genes, and GWAS studies do not appear to indicate imprinted genes are any more likely to be associated with these major neuropsychiatric disorders than any other gene within the genome [58] (although GWAS may not be optimised to detect imprinted genes linked to disorders [59, 60]).

However, studies of rare copy number variants (CNVs) have highlighted strong links between imprinted genes and the incidence of severe neuropsychiatric disorders. Again, these centre on 15q11-q13, but unlike the deletions that lead to AS and PWS (Fig. 1), here the mutations are duplication CNVs that span the imprinted interval. Specifically, an increased incidence of psychotic illness was found in individuals carrying a maternal 15q11-q13 duplication CNV, but not a paternal duplication CNV [61]. Initial studies determined the increased risk in maternal duplication CNV carriers by examining inherited mutations in trios of parent and affected offspring [62]. However, given the nature of the study, the instances of CNVs were low, particularly for paternal derived duplications. By applying methods that examined the pattern of DNA-me on chromosome 15 [63], it was possible to greatly extend this original study and include individuals for which there was no parental DNA sample and/or carrying de novo mutations. This study, which included 28,138 schizophrenia cases, 51,001 developmental delay/autism spectrum disorder cases, and 149,780 controls found that paternal duplications were indeed more rare, but that it was only the maternal duplications that increased the risk of developing schizophrenia. This suggests that an increased dosage of maternally expressed imprinted genes within the 15q11-q13 imprinted cluster predisposes to psychotic illness [64]. This was further underlined by the observation of generation skipping of psychotic illness in families where the duplication was inherited (just over 50% were de novo mutations).

The idea that increased dosage of maternally expressed imprinted genes within the 15q11-q13 imprinted cluster predisposes to psychotic illness is also supported by the observation that some PWS genotypes are more likely to develop psychotic illness than others. As outlined above PWS is caused by loss of paternal imprinted gene expression at 15q11-q13. Most commonly this results as a consequence of a de novo deletion on the paternal chromosome 15 that spans the imprinted interval. However, both maternal chromosome 15 uniparental disomy and a paternal PWS-ICR deletion can also lead to loss of paternal imprinted gene expression but, unlike deletion mutations, these two genotypes also result in 2-fold over-expression of maternal imprinted genes. In addition to the core neurodevelopmental and behavioural phenotypes, individuals with PWS caused by maternal chromosome 15 uniparental disomy or a paternal PWS-ICR deletion are far more likely to develop psychotic illness [65, 66].

We have recently investigated the molecular bases of psychotic illness in PWS by examining mouse models that mimic the critical interval deletion (PWS-cr) and PWS ICR deletion (PWS imprinting centre deletion, or PWS-IC) genotypes. The PWS-cr mice have loss of paternally expressed genes critical to the core phenotype of PWS, and the PWS-IC lose all paternal gene expression and also have 2-fold overdosage of maternal *Ube3a*. These two models both display the core phenotypes associated with PWS, including growth retardation [32, 33, 67], endocrine changes [32, 68], and hyperphagia [31, 69, 70]. However, the PWS-IC mouse model also displays a range of cognitive and behavioural deficits that are endophenotypes for psychiatric illness [67], which are absent in the PWS-cr model [71]. Having established these behavioural differences we then examined brain gene expression using RNA-seq. Mirroring the behavioural findings, there were a number of shared gene changes between the two models, but the PWS-IC mouse also had a further 101 gene expression and splice variant differences. When these 101 genes were interrogated for enrichment of variants associated with psychiatric illness we demonstrated an over-representation of genes linked to single and multiple episodes of psychotic illness, but *not* schizophrenia [71]. This echoes the observation from patients with PWS [72], and indeed those with maternal duplication CNVs affecting 15q11-q13 [62], that indicates their psychotic illness is distinct from schizophrenia.

## Indirect action of imprinted genes on brain and behaviour

As mentioned above, imprinted genes are also concentrated in a number of other tissues outside of the brain, and altered expression in these tissue can lead to indirect effects on brain and behaviour. Of particular relevance here are the large number of imprinted genes that influence the development and/or function of the placenta [73, 74].

The placenta, which is derived from the foetus, has a number of different functions. Possibly most prominent is the transport of nutrients from the mother to the foetus. Demand for nutrients is determined by the foetus and placenta, and can place a considerable burden on maternal resources during pregnancy. Disruption to placental nutrient transport, either through poor maternal diet or stress during pregnancy, is recognised as a key contributor to programming of neurodevelopmental disorders, such as schizophrenia and affective disorders, in the offspring [75]. Although the original attention was *in utero* growth restriction (IUGR) resulting from extreme poor maternal diet as the consequence of famine [76, 77], there is increasing evidence that overprovision by the mother can alter neurodevelopment in the offspring too [78].

Nutrient transport is a key focus for the action of imprinted genes in placenta and disruption to their expression can lead to over- and under-growth of the developing foetus [79, 80]. An important question therefore is whether abnormal expression of these imprinted genes in the placenta can also have a knock-on effect for brain development. Strong evidence for this being the case comes from studies investigating the maternally silenced/paternally expressed *Igf2* gene, which encodes the insulin-like growth factor 2. This gene produces a number of different transcripts, the majority of which are expressed in both the placenta and the foetus (P2 and P3, with P1 expressed in liver) [81]. However, the *Igf2*-P0 transcript is exclusive to the placenta and when deleted in mice leads to IUGR as consequence of an imbalance between foetal demand and supply of nutrients [82]. Animals that lack *Igf2-*P0 also show changes in a range of anxiety related behaviours and brain expression of key GABA_A_ subunit and serotonin receptor genes as adults [83]. This indicates that altered expression of key placental imprinted genes can indeed programme offspring brain and behaviour with consequences that last into adulthood. Interestingly, there is some correlative evidence that changes in DNA-me at the *IGF2* locus may underlie differences in human population exposed to famine in utero [84].

In addition to nutrient transport, the placenta is also an endocrine organ that releases hormones into the maternal circulation, influencing metabolism and foetal growth [85]. One group of hormones are the placental lactogens, which bind to prolactin receptors. Prolactin, which is secreted by the pituitary, and placental lactogens play a critical role during pregnancy, preparing the mammary gland for lactation and priming parental care behaviour in the mother [86]. A number of imprinted genes have now been shown to regulate the development of these endocrine cell lineages in the placenta, thus potentially altering placental lactogen signalling to the mother [73]. A 2-fold increase of expression of one of these imprinted genes, *Phlda2*, in the mouse foetus leads to a 50% decrease in the size of the placental endocrine compartment; conversely, loss of *Phlda2* expression results in a doubling in size of the endocrine compartment [87]. Both these manipulations of *Phlda2* expression in the foetal placenta have consequences for gene expression in the maternal brain and parental care behaviour in the early postnatal phase [88]; this in turn can have knock-on consequences for offspring brain and behaviour later in life [89].

Although not as striking, altered maternal anxiety has been demonstrated for dams carrying fetuses null for another imprinted gene that plays a role in the placental endocrine compartment, namely *Peg3* [90]. Decreased placental expression of *PEG3* has also been linked to perinatal depression in mothers [91]. Although again this is tentative, correlative evidence for a role in humans, the link between imprinted genes, placental function and maternal mental health is an exciting area for future study [92].

## Why should imprinted genes be involved in brain function at all?

Since it was first recognised, and indeed before the initial identification of an actual imprinted gene [93], biologists have speculated as to why imprinting has evolved. Primarily this is because the act of silencing a perfectly functional copy negates the benefits of diploidy [94] and, as I have outlined above, means that mutations in the active parental copy leaves individuals vulnerable to debilitating disorders. This possibly explains the small number of imprinted genes, but also implies that there must be a strong selective pressure leading to the evolution and maintenance of this epigenetic phenomena at the ~200 known canonical imprinted loci [10]. Although there have been many different ideas proposed to explain genomic imprinting [95], two theories have risen to the fore. The original evolutionary theory of genomic imprinting suggested that imprinting arose as a consequence of conflict between maternal and paternal genomes where they have antagonistic ‘interests’ over aspects of physiology [93, 96]. This intragenomic conflict can arise through kinship mechanisms [10] as a consequence of asymmetries of relatedness, as occurs in utero where, unlike the foetal maternal genome, the paternal genome is not shared with the mother [97], and additionally when there is sex-biased dispersal from a social group [98, 99]. More recently differential reproductive variance between males and females has also been invoked to describe how imprinted genes may influence risk taking and impulsive behaviours [100]. In contrast, others have argued that the role of imprinting is to enhance co-adaption by coordinating traits expressed by interacting kin. This idea was originally developed in light of the findings of that some imprinted genes are involved maternal care [101], but has now been expanded to include other aspects of physiology and behaviour [102].

There have been attempts to understand neurodevelopmental and neuropsychiatric disorders linked to imprinted genes in the context of the evolution of imprinting, particularly in terms of intragenomic conflict. One proposal that attempts to explain the role of imprinted genes in the brain generally (called the “imprinted brain” hypothesis) is that the parental genomes are in conflict over the degree of mentalizing cognition, with maternal genes promoting hyper-, and paternal genes promoting hypo-mentalizing [103]. This idea is largely based on the distinct, and sometime oppositional characteristics of AS and PWS [104]. Specifically, the fact that AS is thought of as an autism spectrum disorder (ASD being hypo-mentalism, due to loss of maternally expressed genes), whereas individuals with PWS are more prone to psychotic illness (psychosis being hyper-mentalism, due to loss of paternal expressed genes). However, as I have outlined above, this distinction is not so clearcut [105]. Furthermore, findings since this idea weas proposed showing that maternal duplication CNVs at 15q11-q13 can give rise to ASD and/or psychotic illness [61, 62, 106], suggest that this idea might be oversimplistic.

Others have endeavoured to describe how discrete aspects of the AS and PWS phenotypes may provide insight into the conflict between maternal and paternal genomes over resources from the mother. One example includes the suggestion that the differences in sleep disturbances seen in AS (increased night-waking) and PWS (sleepiness in infants) represent conflict between paternal and maternal genomes over the duration of the mothers’ lactactional amenorrhea and the delay in conception and birth of a subsequent sib [107]. This increased demand on the mother in individuals with AS can also potentially be seen in their ‘happy disposition’ which is more often directed towards primary care-givers [108]. An analogous affect on attention-getting is also seen in a mouse model for AS that have increased ultrasonic vocalisations (USVs), which are used to signal to their mothers and elicit care-giving [109]. Fascinatingly, a recent study of mice lacking the PWS gene *Magel2* demonstrated the opposite phenotype; decreased USVs that led to reduced caregiving from the dam [110].

## Conclusions

Although representing a small subset of the mammalian genome, a number of imprinted genes are expressed in the brain and play critical roles in its development and function. Mutations affecting the expression of these genes, often deletion or duplication CNV mutations, can lead to key defined neurodevelopmental disorders such as Prader-Willi and Temple syndromes, but are also contributors to neuropsychiatric illness more generally. As our understanding of the role of imprinting in neurodevelopmental and neuropsychiatric disorders increases, so researchers can begin to develop targeted genetic and/or epigenetic therapeutic strategies that may restore imprinted gene expression to normal levels, and potentially rescue any deficits.

In addition, imprinted genes are strongly over-represented in the placenta, and changes in their expression here may also have consequences for brain and behaviour disorders in both the offspring and the mother. This may occur via an imbalance in the supply and demand of nutrients for the foetus via the placenta; or by changes in the endocrine signalling from the placenta to the mother. This may be an important developing area for imprinted gene research and brain function, reflecting a wider recognition that genes expressed in the placenta can influence the aetiology of neuropsychiatric illness [111].
